# Mechanisms of hyperinsulinaemia in apparently healthy non-obese young adults: role of insulin secretion, clearance and action and associations with plasma amino acids

**DOI:** 10.1007/s00125-019-04990-y

**Published:** 2019-09-06

**Authors:** Steven Hamley, Danielle Kloosterman, Tamara Duthie, Chiara Dalla Man, Roberto Visentin, Shaun A. Mason, Teddy Ang, Ahrathy Selathurai, Gunveen Kaur, Maria G. Morales-Scholz, Kirsten F. Howlett, Greg M. Kowalski, Christopher S. Shaw, Clinton R. Bruce

**Affiliations:** 1 Institute for Physical Activity and Nutrition, School of Exercise and Nutrition Sciences, Deakin University, 221 Burwood Highway, Burwood, VIC 3125 Australia; 2 Department of Information Engineering, University of Padova, Padova, Italy

**Keywords:** Hyperinsulinaemia, Insulin secretion, Insulin sensitivity, Minimal model, Plasma amino acids, Prediabetes

## Abstract

**Aims/hypothesis:**

This study aimed to examine the metabolic health of young apparently healthy non-obese adults to better understand mechanisms of hyperinsulinaemia.

**Methods:**

Non-obese (BMI < 30 kg/m^2^) adults aged 18–35 years (*N* = 254) underwent a stable isotope-labelled OGTT. Insulin sensitivity, glucose effectiveness and beta cell function were determined using oral minimal models. Individuals were stratified into quartiles based on their insulin response during the OGTT, with quartile 1 having the lowest and quartile 4 the highest responses.

**Results:**

Thirteen per cent of individuals had impaired fasting glucose (IFG; *n* = 14) or impaired glucose tolerance (IGT; *n* = 19), allowing comparisons across the continuum of insulin responses within the spectrum of normoglycaemia and prediabetes. BMI (~24 kg/m^2^) was similar across insulin quartiles and in those with IFG and IGT. Despite similar glycaemic excursions, fasting insulin, triacylglycerols and cholesterol were elevated in quartile 4. Insulin sensitivity was lowest in quartile 4, and accompanied by increased insulin secretion and reduced insulin clearance. Individuals with IFG had similar insulin sensitivity and beta cell function to those in quartiles 2 and 3, but were more insulin sensitive than individuals in quartile 4. While individuals with IGT had a similar degree of insulin resistance to quartile 4, they exhibited a more severe defect in beta cell function. Plasma branched-chain amino acids were not elevated in quartile 4, IFG or IGT.

**Conclusions/interpretation:**

Hyperinsulinaemia within normoglycaemic young, non-obese adults manifests due to increased insulin secretion and reduced insulin clearance. Individual phenotypic characterisation revealed that the most hyperinsulinaemic were more similar to individuals with IGT than IFG, suggesting that hyperinsulinaemic individuals may be on the continuum toward IGT. Furthermore, plasma branched-chain amino acids may not be an effective biomarker in identifying hyperinsulinaemia and insulin resistance in young non-obese adults.

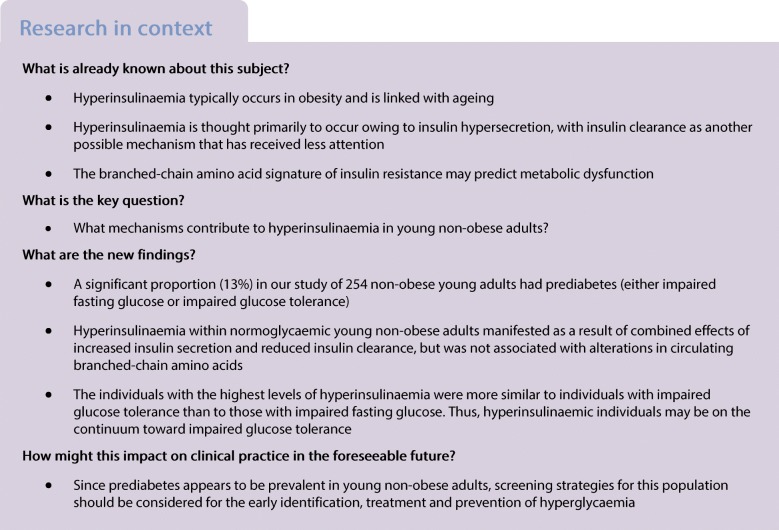



## Introduction

Hyperinsulinaemia is the central component of the metabolic syndrome, and is an independent predictor of metabolic and cardiovascular disease [1–6]. Classically, hyperinsulinaemia is viewed as a compensatory response whereby beta cells hypersecrete insulin to overcome reduced tissue insulin action to maintain normal glycaemic control. However, it is not clear how, in the face of normal blood glucose, beta cells sense and adjust insulin secretion to precisely compensate for tissue insulin resistance [7]. An alternative hypothesis exists whereby primary insulin hypersecretion initiates insulin resistance [8–10]. In this scenario, hyperinsulinaemia downregulates tissue insulin action (insulin-induced insulin resistance) [8–13]. Regardless of its evolution, hyperinsulinaemia is pathological; thus, understanding what drives hyperinsulinaemia is of high importance.

Hyperinsulinaemia can develop due to the interplay among the degree of tissue insulin action, beta cell insulin secretion, plasma glucose levels and the rate of insulin clearance. The widely held view is that hyperinsulinaemia is an obesity-related phenomenon, although it should be noted that not all obese individuals exhibit hyperinsulinaemia [14]. In addition to insulin hypersecretion, insulin clearance is reduced in obese insulin-resistant individuals [15, 16] and may be an additional mechanism that contributes to hyperinsulinaemia. However, it is not clear whether reduced insulin clearance also contributes to hyperinsulinaemia independent of obesity.

An additional risk factor for hyperinsulinaemia is ageing [17, 18]. However, insulin resistance and hyperinsulinaemia are evident in children and young adults [19]. It is therefore critical to diverge from the predominantly obesity-centric and ageing-related viewpoint of hyperinsulinaemia and insulin resistance. Hence, the aim of this study was to improve our understanding of the hyperinsulinaemic insulin-resistant state by examining the metabolic health of apparently healthy non-obese young adults. Non-obese apparently healthy young adults underwent a 3 h OGTT enriched with isotopically labelled glucose and were stratified based on their integrated insulin responses. Oral glucose and C-peptide minimal models were used to determine indices of insulin action on glucose disposal and production [20–22], beta cell insulin secretion [23] and insulin clearance. Additionally, the emergence of the branched-chain amino acid (BCAA) signature of insulin resistance [24–26] suggests that amino acids may be involved in the pathogenesis of hyperinsulinaemia. Therefore, targeted quantitative plasma amino acid profiling was performed to further evaluate the relationship between hyperinsulinaemia and amino acids.

## Methods

### Participants

The Deakin University Human Research Ethics Committee approved this study. In total, 254 healthy individuals (151 women; 103 men) were recruited from the university and surrounding area via advertisement on campus and word of mouth. Informed, written consent was obtained prior to participation. Individuals had to have a BMI < 30 kg/m^2^, be 18–35 years old with no previous diagnosis of diabetes or prediabetes (i.e. impaired fasting glucose [IFG] or impaired glucose tolerance [IGT]) and not taking medications known to affect metabolism.

### Experimental procedures

Food diaries were recorded for two weekdays and one weekend day prior to the study for assessment of energy and macronutrient intake (FoodWorks, Brisbane, QLD, Australia). Individuals refrained from physical activity for 48 h prior to study. Upon arrival at the laboratory (08:00–09:00 h) following an overnight fast (~10 h), height and weight were recorded and body composition assessed by dual-energy X-ray absorptiometry (Lunar Prodigy, GE Medical Systems, Madison, WI, USA). From a forearm vein catheter (22 gauge), baseline (−10 and 0 min) bloods (3 ml) were collected before individuals consumed a drink containing 75 g glucose (Daniels Health, Dandenong South, VIC, Australia) enriched with [6,6-^2^H]glucose (4% wt/vol.; Cambridge Isotope Laboratories, Tewksbury, MA, USA). Blood was sampled at 10, 20, 30, 60, 90, 120, 150 and 180 min, immediately placed on ice, later spun in a centrifuge and plasma stored at −80°C.

### Analytical techniques

Plasma glucose was determined using the glucose oxidase method. Plasma NEFA (Wako Chemicals, Richmond, VA, USA), triacylglycerols (Roche Diagnostics, Indianapolis, IN, USA), and total cholesterol (Wako Chemicals) and HDL-cholesterol (Crystal Chem, Elk Grove Village, IL, USA) were determined using enzymatic assays. Plasma insulin (ALPCO, Salem, NH, USA) and C-peptide (Millipore, Burlington, MA, USA) were determined by ELISA. GC-MS was used to determine plasma [6,6-^2^H]glucose enrichment [27]. Plasma amino acid analysis was performed as previously described [28].

### Calculations and statistics

Thirteen percent of individuals (*n* = 33) with prediabetes were identified and classified as having IFG (fasting glucose 6.1–7.0 mmol/l; *n* = 14) or IGT (2 h OGTT glucose 7.8–11.1 mmol/l; *n* = 19). As no definitive criteria exist to classify hyperinsulinaemia, participants were stratified into quartiles (Q) based on their integrated insulin response determined by calculating the area above basal (AAB) for the insulin concentration curve during the OGTT. With this approach, Q1 had the lowest and Q4 the highest insulin response. Individual characteristics of each quartile were compared with those classified as having IFG or IGT, thereby allowing comparison along the continuum of insulin responses within the spectrum of normoglycaemia and prediabetes. Fasting hormones and metabolites are reported as the mean of baseline samples. Indices of insulin sensitivity (S_I_) and glucose effectiveness (GE), the ability of insulin and glucose, respectively, to stimulate glucose disposal (S_I_^D^/GE^D^) and inhibit glucose production (S_I_^L^/GE^L^), were determined using the single-tracer oral glucose minimal model [20–22]. These indices were estimated from plasma glucose tracer and insulin concentrations as previously described [20–22]. Beta cell responsivity indexes were estimated from plasma glucose and C-peptide concentrations during the OGTT using the oral C-peptide minimal model [23]. Beta cell function was assessed in terms of basal (Φ_b_), static (Φ_s_), dynamic (Φ_d_) and total (Φ_tot_) responsivity indices [23]. The disposition index (DI) (an assessment of insulin secretion in relation to the prevailing degree of S_I_) was calculated by multiplying Φ_tot_ by total S_I_. Insulin secretion rate (ISR) was calculated from C-peptide using Insulin SECretion software [29] which was kindly provided by R. Hovarka (University of Cambridge, Cambridge, UK). Insulin clearance was calculated as the ratio of the AAB for insulin secretion to the insulin concentration AAB. Data were analysed using one-way ANOVA followed by Tukey’s multiple comparison test. For non-normally distributed data, Kruskal–Wallis tests were used followed by Dunn’s multiple comparison test. Data are reported as mean ± SEM if normally distributed, and as median (interquartile range) for variables with a skewed distribution (D’Agostino–Pearson test). Statistical significance was accepted when *p* < 0.05.

## Results

### Participant characteristics

Characteristics of each insulin response quartile as well as of those with IFG and IGT are shown in Table 1. All groups were similar in terms of age; however, individuals in Q1 were taller than those in Q2–4 and with IGT and heavier than those in Q2–4. Individuals with IFG were also taller than individuals in Q4 and with IGT, likely because of a greater proportion of men in both Q1 and the IFG group (*χ*^2^ test for sex *p* < 0.0001). While BMI was similar across groups, the proportion of overweight (BMI > 25 kg/m^2^) individuals was significantly different among groups (*χ*^2^ test *p* < 0.01), with a greater proportion in the IGT group. Individuals in Q4 and with IGT had the highest relative and absolute fat mass, while fat-free mass was highest in individuals in Q1 and with IFG. Visceral fat was similar for all groups. Systolic BP was similar across groups while diastolic BP was higher in individuals in Q4 and with IGT than in those in Q2. Compared with Q1 and Q2, fasting triacylglycerols were elevated in Q4 and in those with IGT. Total cholesterol was higher in Q4 compared with Q1 and Q2. HDL was similar across groups; however, individuals in Q4 had higher levels of non-HDL-cholesterol compared with Q1. Family history of diabetes (up to first-degree relatives and grandparents) was significantly different (χ^2^ test *P*< 0.001), with Q3, Q4 and IFG exhibiting the highest proportion with a positive family history. A statistically significant difference was also found in ethnicity (*χ*^2^ test *p* < 0.0001), with a greater proportion of Asians in Q3, Q4 and the IGT group.

**Table 1:** Participant characteristics

Characteristic	Insulin AUC quartile				IFG	IGT
	Q1	Q2	Q3	Q4		
*n* (F/M)	55 (15/40)	56 (38/18)	55 (40/15)	55 (37/18)	14 (6/8)	19 (15/4)
Age (years)	24 (6)	24 (5)	22 (5)	24 (6)	22 (3)	24 (7)
Height (cm)	179 (11)	169 (12)*	168 (10)*	167 (16)*^¶^	177 (10)	166 (9)*^¶^
Weight (kg)	76.2 (15.8)	68.2 (17.1)*	62.1 (16.7)*	64.9 (18.8)*	74.5 (21.0)	62.8 (18.6)
BMI (kg/m^2^)	24.1 (3.5)	23.4 (3.8)	22.3 (3.8)	23.8 (5.7)	23.6 (3.4)	24.9 (5.1)
BMI > 25 kg/m^2^ (%)	31	30	24	34	21	47
Body fat (%)^a^	19.1 ± 1.1	25.9 ± 1.0*	28.9 ± 1.1*	32.1 ± 1.4*^†^	25.4 ± 2.6	32.2 ± 1.5*^†^
Fat mass (kg)	12.7 (7.0)	16.8 (6.1)	17.2 (7.0)*	20.8 (9.0)*	18.0 (5.8)	23.2 (7.3)*
Visceral fat (g)	221 (255)	97 (157)	81 (168)	162 (168)	174 (193)	185 (355)
Fat-free mass (kg)	59.4 (15.0)	46.8 (13.7)*	42.5 (9.5)*	42.0 (12.7)*	57.0 (21.2)	39.9 (8.7)*
SBP	120 (15)	119 (17)	121 (18)	120 (18)	125 (13)	120 (12)
DBP	73 (11)	73 (11)	77 (10)	80 (13)^†^	71 (13)	80 (8)^†^
Fasting glucose (mmol/l)	5.4 ± 0.1	5.4 ± 0.1	5.3 ± 0.1	5.3 ± 0.1	6.3 ± 0.1*^†‡§^	5.5 ± 0.1^¶^
2 h glucose (mmol/l)	4.7 (1.5)	5.0 (1.4)	5.0 (1.1)	5.8 (1.9)*^†^	5.6 (1.0)	8.8 (1.4)*^†‡§¶^
Fasting insulin (pmol/l)	10.6 (8.9)	17.5 (11.6)	20.0 (15.8)*	31.5 (22.9)*^†‡^	22.1 (11.3)*	30.3 (23.4)*^†^
2 h insulin (pmol/l)	51.5 (60.8)	106.0 (67.8)*	156.8 (73.5)*^†^	261.5 (168.8)*^†‡^	114.9 (114.8)*^§^	355.0 (314.7)*^†‡¶^
Fasting C-peptide (pmol/l)	199.3 (101.3)	267.4 (153.9)*	289.9 (147.8)*	322.1 (214.7)*	339.1 (121.0)*	351.0 (222.5)*
Fasting NEFA (mmol/l)	0.20 (0.15)	0.23 (0.15)	0.26 (0.17)	0.22 (0.21)	0.17 (0.19)	0.35 (0.15)*
Fasting triacylglycerol (mmol/l)	0.63 (0.25)	0.59 (0.25)	0.73 (0.22)	0.86 (0.29)*^†^	0.81 (0.39)	0.94 (0.40)*^†^
Total cholesterol (mmol/l)	3.9 (0.8)	3.9 (1.0)	4.2 (1.0)	4.5 (1.0)*^†^	4.1 (0.9)	4.3 (1.1)
HDL-cholesterol (mmol/l)	1.4 (0.4)	1.4 (0.4)	1.5 (0.4)	1.5 (0.5)	1.4 (0.3)	1.5 (0.6)
Non-HDL-cholesterol (mmol/l)	2.4 (0.7)	2.4 (0.8)	2.7 (0.9)	2.8(1.4)*	2.7 (0.9)	2.9 (0.8)
Family history of diabetes (%)	16	20	35	31	36	5
Smokers (%)	5	4	2	4	0	5
Energy intake (MJ)^b^	10.9 (4.0)	8.7 (2.5)*	8.1 (2.4)*	7.5 (2.8)*	10.9 (3.0)	7.2 (3.4)*
% carbohydrate	38.3 (14.0)	40.9 (11.1)	38.8 (9.1)	42.0 (8.8)	35.9 (8.5)	38.3 (8.7)
% protein	20.7 (5.2)	18.0 (5.7)	19.5 (5.8)	18.4 (4.0)	23.0 (7.2)	21.9 (8.1)
% fat	34.1 (9.9)	34.1 (10.1)	36.7 (8.5)	33.4 (9.1)	37.7 (3.4)	36.6 (5.3)
Contraceptive use (% women)	7	11	23	30	33	33
White (%)	94	84	76	62	93	74
Asian (%)	4	16	24	34	7	26
Other (%)	2	0	0	4	0	0

Data are mean ± SEM, median (interquartile range) or proportion (%) as appropriate

^a^Data on body composition (percentage fat, fat mass, visceral fat and lean mass) are from *n* = 45 for Q1, *n* = 40 for Q2, *n* = 43 for Q3, *n* = 30 for Q4, *n* = 9 for IFG and *n* = 17 for IGT

^b^Data on energy and macronutrient intake are from *n* = 40 for Q1, *n* = 45 for Q2, *n* = 42 for Q3, *n* = 34 for Q4, *n* = 10 for IFG and *n* = 12 for IGT

*p < 0.05 vs Q1

^†^*p* < 0.05 vs Q2

^‡^*p* < 0.05 vs Q3

^§^*p* < 0.05 vs Q4

^¶^*p* < 0.05 vs IFG

F/M, female/male

### Plasma glucose, insulin, C-peptide, ISR and NEFA

**Fig. 1:**
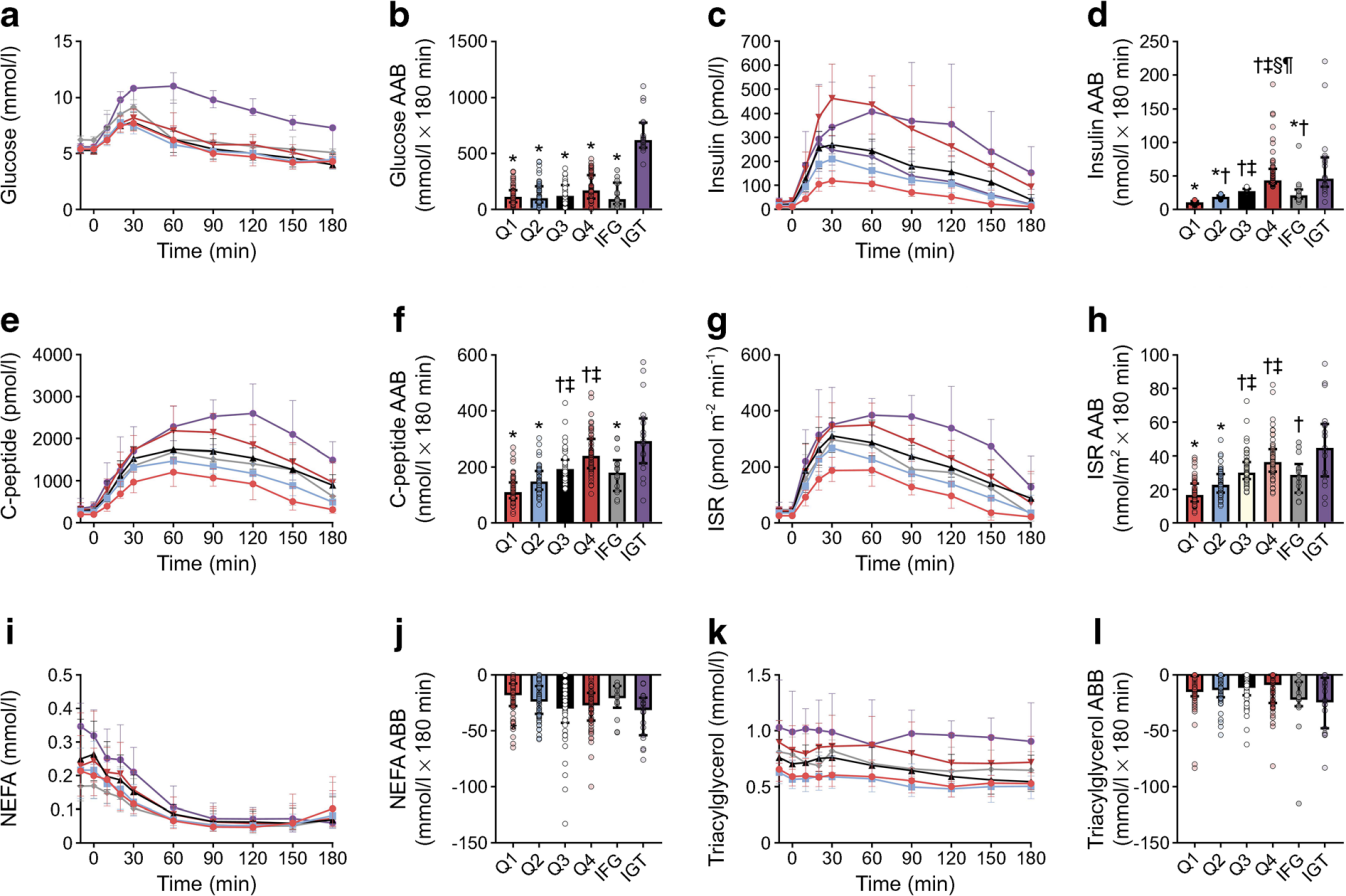
Plasma metabolite and hormone concentrations during the OGTT, which began at time 0 min. (**a**) Plasma glucose concentrations. (**b**) The AAB for the glucose response. (**c**) Plasma insulin concentrations. (**d**) The AAB for the insulin response. (**e**) Plasma C-peptide concentrations. (**f**) The AAB for the C-peptide response. (**g**) ISR. (**h**) The AAB for the ISR response. (**i**) Plasma NEFA concentrations. (**j**) The area below basal (ABB) for the NEFA response. (**k**) Plasma triacylglycerol concentrations. (**l**) The ABB for the triacylglycerol response. Pink circles, Q1; blue squares, Q2; black triangles, Q3; red triangles, Q4; grey diamonds, IFG; purple circles, IGT. Data are median and interquartile range. *p< 0.05 vs IGT; ^†^*p*< 0.05 vs Q1; ^‡^*p*< 0.05 vs Q2; ^§^*p*< 0.05 vs Q3; ^¶^*p*< 0.05 vs IFG

Fasting glucose was higher in individuals with IFG compared with all other groups (Table 1) but was similar across Q1 to Q4 and in those with IGT. Glucose responses during the OGTT were similar for all quartiles (Fig. 1a, b). However, within the normoglycaemic range, 2 h glucose was higher in participants in Q4 compared with Q1 and Q2 as well in those with IFG when compared with Q1 (Table 1). The glucose AAB during the OGTT in the IFG individuals was not different from individuals in Q1–4 (Fig. 1b). Compared with all other groups, individuals with IGT exhibited a higher peak and 2 h glucose (Table 1), as well as a greater AAB (Fig. 1b).

**Fig. 2:**
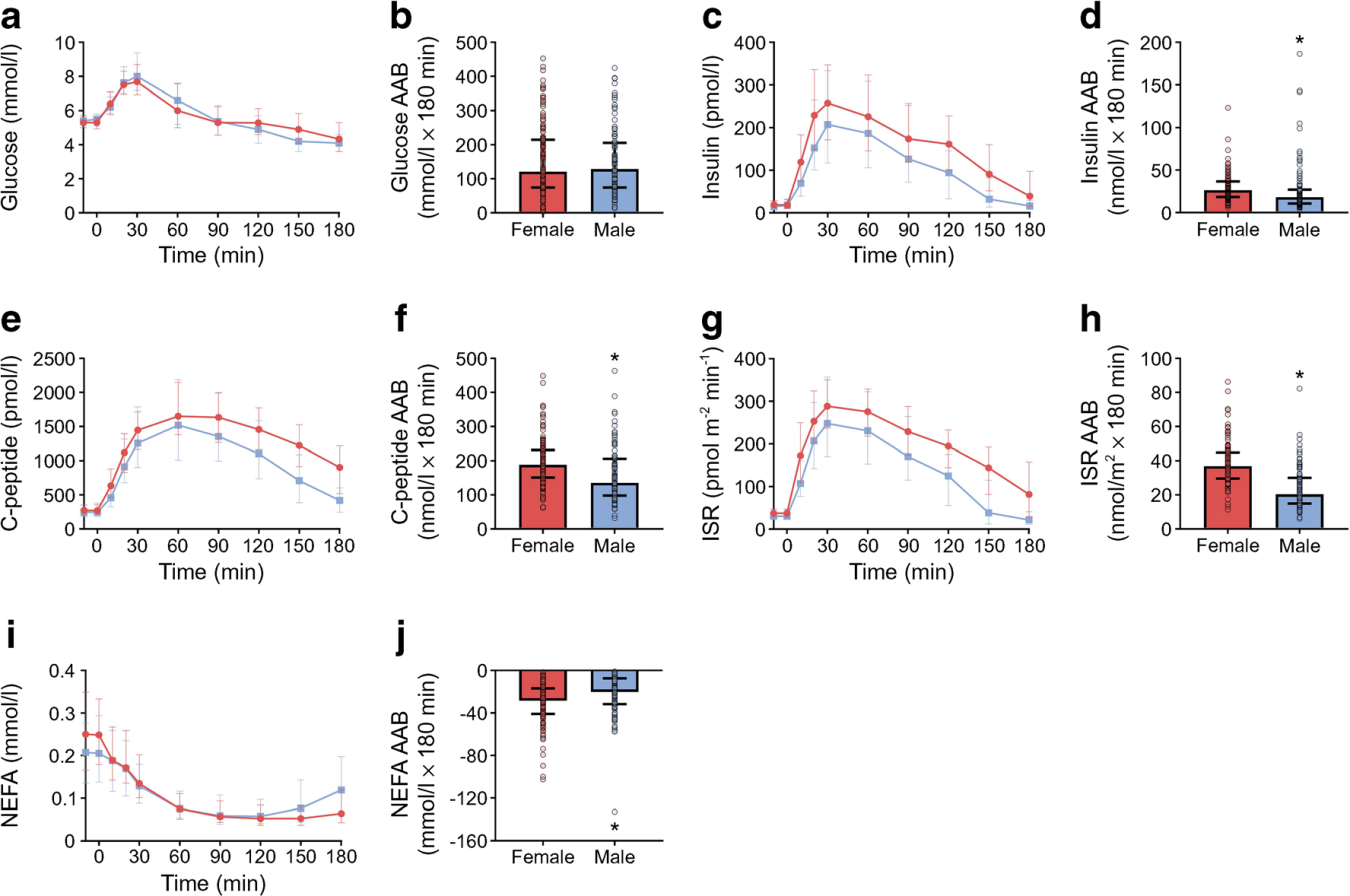
Metabolic responses during the OGTT in women vs men. The OGTT began at time 0 min. (**a**) Plasma glucose concentrations. (**b**) The AAB for the glucose response. (**c**) Plasma insulin concentrations. (**d**) The AAB for the insulin response. (**e**) Plasma C-peptide concentrations. (**f**) The AAB for the C-peptide response. (**g**) ISR. (**h**) The AAB for the ISR response. (**i**) Plasma NEFA concentrations. (**j**) The area below basal (ABB) for the NEFA response. Red circles, women; blue squares, men. Data are median and interquartile range. *p< 0.001 vs women

Fasting insulin was lowest in Q1 and progressively rose across quartiles, with Q4 having significantly higher fasting insulin than Q1–3 (Table 1). Individuals with IFG had higher fasting insulin compared with Q1 only. Compared with Q1 and Q2, fasting insulin was elevated in individuals with IGT (Table 1). By design, the insulin responses during the OGTT progressively increased across quartiles (Fig. 1c,d) and a similar pattern was also observed for 2 h insulin (Table 1). The insulin response in individuals with IFG was intermediate, with the AAB (Fig. 1d) and 2 h insulin levels (Table 1) higher than Q1 yet lower than Q4 and IGT. In contrast, individuals with IGT had the highest 2 h insulin levels of any group (Table 1), yet the insulin AAB was comparable to that of Q4 (Fig. 1d). Individuals with IGT also exhibited a delayed insulin response, with peak levels reached at 60 min, which contrasts with other groups where insulin peaked at or before 30 min (Fig. 1c).

Compared with Q1, fasting C-peptide was higher in all other groups (Table 1). During the OGTT, Q3 and Q4 exhibited greater C-peptide responses than both Q1 and Q2 (Fig. 1e, f). Except for Q3 and Q4, the C-peptide AAB was higher in individuals with IGT compared with all other groups (Fig. 1f). Similarly, ISRs were higher in Q3 and Q4 when compared with Q1 and Q2 (Fig. 1g, h). The AAB for insulin secretion was also higher in individuals with IGT than in individuals in Q1 and Q2 (Fig. 1h). The insulin secretion response in individuals with IFG was only different from that of Q1 where it was significantly elevated.

Fasting NEFA was higher in individuals with IGT than in those in Q1, as was the magnitude of NEFA suppression during the OGTT (Fig. 1i, j, Table 1). The triacylglycerol response during the OGTT was similar among groups (Fig. 1k, l).

### Indices of S_I_ and GE

Across the quartiles, there was a progressive reduction in S_I_^D^, S_I_^L^ and total S_I_ (Table 2). While S_I_^D^ and total S_I_ were lower in individuals with IFG compared with Q1, S_I_^L^ was similar (Table 2). S_I_ was similar between the IFG group and Q2 and Q3, yet when compared with Q4, individuals with IFG had greater S_I_^L^ and total S_I_ (Table 2). S_I_^D^, S_I_^L^ and total S_I_ were lower in individuals with IGT compared with those in Q1–3 and with IFG, but were similar to Q4 (Table 2). Although GE^D^ was lower in individuals with IGT compared with all other groups, GE^L^ was not different (Table 2). Compared with Q1, GE^D^ was also lower in Q4. Total GE was also lower in individuals with IGT than in individuals with IFG and in those in Q2–4 (Table 2).

**Table 2:** S_I_, GE and beta cell function

Variable	Insulin AUC quartile				IFG	IGT
	Q1	Q2	Q3	Q4		
S_I_^D^ (dl^−4^ kg^−1^ min^−1^ [pmol/l]^−1^)	263.5 (225.9)	155.4 (109.6)*	123.0 (88.1)*	64.1 (60.1)*^†‡^	113.5 (61.9)*	19.8 (27.2)*^†‡¶^
S_I_^L^ (dl^−4^ kg^−1^ min^−1^ [pmol/l]^−1^)	190.4 (113.4)	109.9 (81.0)*	68.9 (36.4)*^†^	45.0 (33.3)*^†¶^	108.3 (88.1)	27.3 (28.1)*^†‡¶^
S_I_ (dl^−4^ kg^−1^ min^−1^ [pmol/l]^−1^)	472.0 (272.4)	257.7 (166.8)*	211.3 (94.8)*	113.1 (90.0)*^†‡¶^	263.8 (96.9)*	47.0 (48.6)*^†‡¶^
GE^D^ (dl kg^−1^ min^−1^)	0.027 (0.006)	0.027 (0.006)	0.025 (0.004)	0.024 (0.003)*	0.026 (0.003)	0.022 (0.001)*^†‡§¶^
GE^L^ (dl kg^−1^ min^−1^)	0.021 (0.025)	0.017 (0.036)	0.025 (0.004)	0.024 (0.003)	0.034 (0.017)	0.015 (0.009)
GE (dl kg^−1^ min^−1^)	0.046 (0.029)	0.045 (0.038)	0.050 (0.025)	0.049 (0.024)	0.059 (0.021)	0.038 (0.009)^†‡§¶^
Φ_b_ (10^−9^ min^−1^)	2.5 (1.2)	3.7 (2.3)*	4.0 (2.2)*	4.4 (3.1)*	3.8 (1.9)	3.9 (1.9)*
Φ_d_ (10^−9^)	216.2 (268.6)	327.9 (301.8)	406.8 (455.1)*	500.9 (440.8)*	415.3 (186.2)	330.6 (406.1)
Φ_s_ (10^−9^ min^−1^)	33.1 (17.2)	38.6 (23.1)	43.2 (29.6)*	51.4 (26.7)*	52.8 (28.5)	31.4 (18.6)^§^
Φ_tot_ (10^−9^ min^−1^)	34.7 (18.6)	40.3 (25.1)	44.7 (30.3)*	52.8 (27.1)*	54.6 (31.5)	34.1 (20.1)^§^
DI (10^−14^ dl^−1^ kg^−1^ min^−2^ [pmol/l]^−1^)	3506 (3443)	2165 (2038)	1854 (1617)*	1083 (1217)*^†^	2143 (2398)	345 (288)*^†‡§¶^
Insulin clearance (l min^−1^ m^−2^)	1.6 (0.9)	1.2 (0.4)*	1.1 (0.5)*	0.7 (0.4)*^†‡¶^	1.2 (0.6)*	0.9 (0.6)*

Data are median (interquartile range)

*p < 0.05 vs Q1

^†^*p* < 0.05 vs Q2

^‡^*p* < 0.05 vs Q3

^§^*p* < 0.05 vs Q4

^¶^*p* < 0.05 vs IFG

### Indices of beta cell insulin secretion and insulin clearance

Individuals in Q2–4 and with IGT exhibited enhanced basal beta cell responsivity (Φ_b_) compared with individuals in Q1 (Table 2). The dynamic component, representing the response to a change in glucose (Φ_d_), was higher in Q4 and Q3 than in Q1 (Table 2). Similarly, the static component denoting the response to a given glucose level (Φ_s_; Table 2) and the overall response to glucose (Φ_tot_; Table 2) were greater in Q4 and Q3 than in Q1. Both Φ_s_ and Φ_tot_ were higher in Q4 than in individuals with IGT. The DI progressively declined across quartiles with the DI of Q3 being lower than Q1, and Q4 was lower than Q1 and Q2 (Table 2). Individuals with IGT exhibited a markedly reduced DI compared with all groups (Table 2). Indices of beta cell function were not altered in IFG. Compared with Q1, insulin clearance was lower in Q2 and Q3 as well as in those with IFG and IGT (Table 2). Individuals in Q4 exhibited reduced insulin clearance in comparison with Q1–3 and the IFG group (Table 2).

### Amino acids

Phenylalanine was elevated in Q4 compared with Q3 (Table 3), while aspartate was elevated in Q4 compared with Q1 and Q2 (Table 3). Plasma glutamate levels were higher in Q4 than in both Q1 and Q3. In contrast, Q4 exhibited lower glutamine levels compared with Q1 (Table 3). Amino acids were not altered in either IFG or IGT (Table 3).

**Table 3:** Plasma amino acid concentrations

Amino acid (μmol/l)	Insulin AUC quartile				IFG	IGT
	Q1	Q2	Q3	Q4		
Alanine	278.2 (56.6)	294.0 (81.4)	285.2 (91.2)	300.8 (91.4)	286.9 (42.4)	311.1 (67.0)
Glycine	227.1 (52.7)	221.4 (58.4)	217.5 (60.3)	229.0 (80.0)	215.5 (45.8)	191.4 (77.7)
Valine	229.9 (61.0)	212.6 (66.2)	206.0 (52.2)	216.8 (69.6)	223.9 (66.6)	229.7 (68.6)
Leucine	126.6 (28.1)	112.5 (31.2)	109.9 (29.9)	119.8 (31.9)	114.8 (30.7)	128.8 (32.7)
Isoleucine	60.5 (15.1)	55.5 (14.7)	53.1 (14.3)	57.8 (16.8)	56.1 (15.7)	64.5 (24.3)
Proline	152.9 (63.5)	160.2 (81.7)	143.0 (46.6)	165.0 (71.6)	176.2 (72.4)	141.8 (59.3)
Methionine	23.4 (6.5)	23.1 (6.5)	22.8 (6.5)	23.7 (5.4)	23.8 (5.8)	22.5 (4.6)
Serine	99.0 (17.2)	100.5 (26.6)	104.3 (26.2)	100.7 (26.8)	95.7 (23.2)	93.6 (10.7)
Threonine	124.7 (46.0)	132.1 (40.2)	130.0 (70.6)	144.9 (70.0)	146.5 (31.4)	137.8 (32.6)
Phenylalanine	59.3 (8.2)	57.9 (12.0)	58.0 (9.6)	62.1 (14.3)^‡^	61.4 (4.2)	63.4 (15.3)
Aspartate	2.0 (4.3)	2.1 (4.1)	2.7 (2.3)	3.8 (5.2)*^†^	2.7 (3.0)	3.0 (2.1)
Glutamate	22.0 (24.0)	28.1 (41.0)	25.3 (26.3)	52.7 (74.9)*^‡^	26.2 (8.7)	50.4 (43.9)
Glutamine	513.6 (118.8)	490.7 (88.2)	482.4 (107.0)	455.2 (88.1)*	488.2 (68.0)	434.1 (66.4)
Tyrosine	61.6 (16.6)	63.9 (22.3)	58.1 (23.3)	65.1 (30.9)	65.4 (16.7)	66.8 (23.9)
Total BCAA	415.1 (107.9)	382.6 (101.0)	369.8 (92.1)	391.9 (126.5)	403.0 (86.4)	427.7 (126.8)

Data are median (interquartile range)

*p < 0.05 vs Q1

^†^*p* < 0.05 vs Q2

^‡^*p* < 0.05 vs Q3

### Sex-specific comparisons

Subgroup analysis was performed on insulin response quartiles for women and men separately, because, first, the sex distribution among quartiles was skewed, with more men in Q1. This likely contributed to differences in body composition among quartiles as men had less fat mass and greater fat-free mass than women (Table 4). Second, women exhibited significantly greater OGTT insulin responses (Fig. 2). This analysis was only performed on normoglycaemic individuals as the IFG and IGT groups were limited by smaller group sizes.

**Table 4:** Participant characteristics of normoglycaemic women vs men

Characteristic	Women	Men
*n*	130	91
Age (years)	23 (5)	24 (7)
Height (cm)	166 (8)	180 (8)*
Weight (kg)	62.5 ± 0.8	80.1 (1.2)*
BMI (kg/m^2^)	22.4 (4.2)	24.6 (4.2)*
BMI > 25 kg/m^2^ (%)	33	40
Body fat (%)^a^	30.4 ± 0.7	18.9 ± 0.8*
Fat mass (kg)	17.8 (8.0)	14.7 (8.8)*
Visceral fat (g)	59.5 (101.0)	250.5 (222.8)*
Fat-free mass (kg)	41.4 (7.5)	59.5 (12.9)*

Data are mean ± SEM, median (interquartile range) or proportion (%) as appropriate

*p < 0.0001

^a^Data on body composition (percentage fat, fat mass, visceral fat and lean mass) are from *n* = 96 for women and *n* = 62 for men

**Fig. 3:**
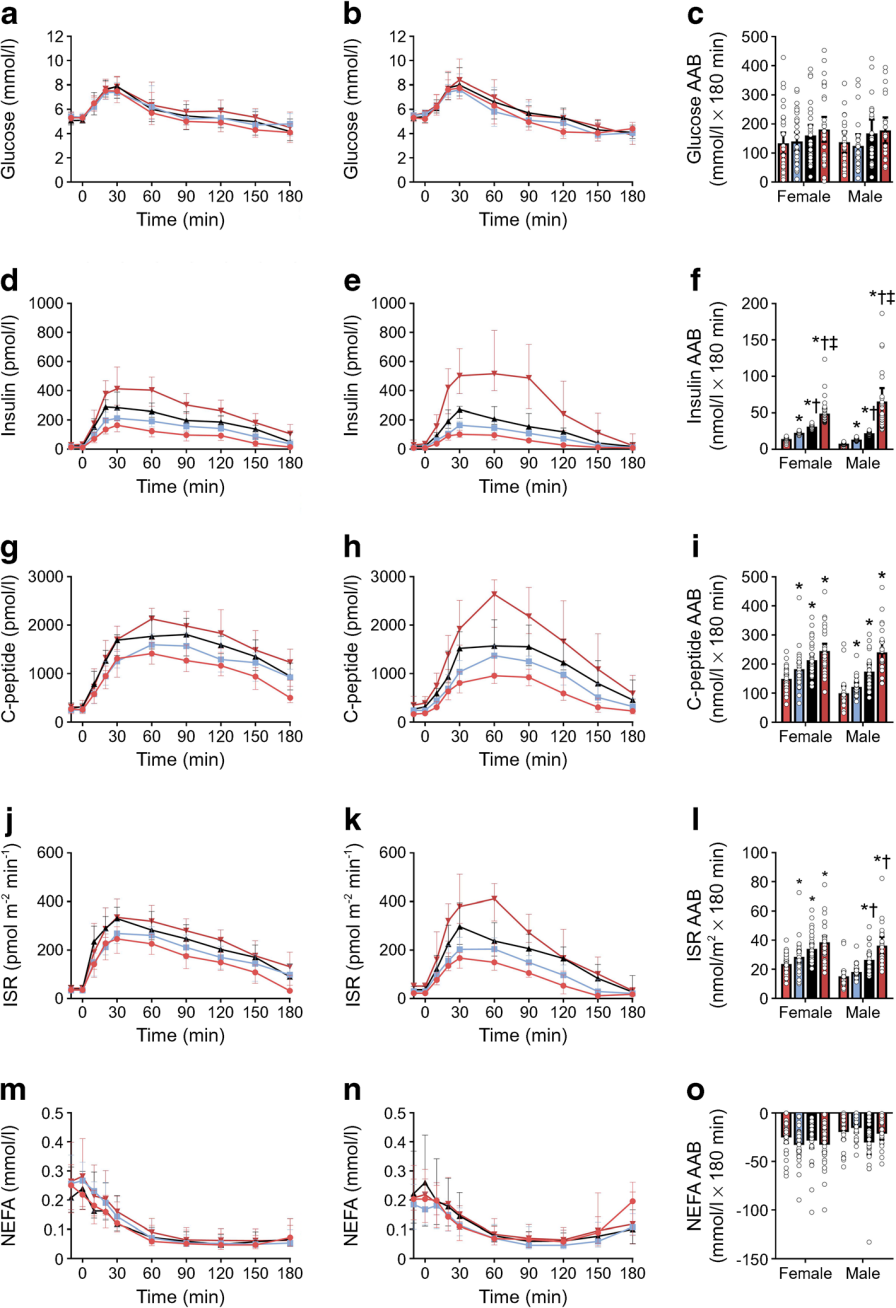
Sex-specific plasma metabolite and hormone concentrations in normoglycaemic women (**a**, **d**, **g**, **j**, **m**) and men (**b**, **e**, **h**, **k**, **n**) during the OGTT. The OGTT began at time 0 min. (**a**, **b**) Plasma glucose concentrations. (**c**) The AAB for the glucose response. (**d**, **e**) Plasma insulin concentrations. (**f**) The AAB for the insulin response. (**g**, **h**) Plasma C-peptide concentrations. (**i**) The AAB for the C-peptide response. (**j**, **k**) ISR. (**l**) The AAB for the ISR response. (**m**, **n**) Plasma NEFA concentrations. (**o**) The area below basal (ABB) for the NEFA response. Pink circles, Q1; blue squares, Q2; black triangles, Q3; red triangles, Q4. Data are median and interquartile range. *p< 0.05 vs Q1; ^†^*p*< 0.05 vs Q2; ^‡^*p*< 0.05 vs Q3

All female quartiles were similar in terms of age, BMI, and body and fat mass (Table 5). However, when compared with Q1, Q4 exhibited greater percentage body fat, while fat-free mass was lower in both Q3 and Q4 (Table 5). There were no differences in plasma lipids, contraceptive use, family history of diabetes and ethnicity (Table 5). While fasting glucose (Table 5) and glucose tolerance (Fig. 3a, c) were similar across quartiles, 2 h glucose was elevated in Q4 vs Q1 (Table 5). There was a progressive increase in both fasting (Table 5) and OGTT insulin responses across quartiles (Fig. 3d, f). However, C-peptide (Fig. 3g, i) and ISR (Fig. 3j, l) were only higher in Q2–4 compared with Q1. All indices of S_I_ were highest in Q1 compared with the other quartiles and were higher in Q2 and Q3 than in Q4 (Table 6). GE^D^ was also lower in Q4 than in Q1, while both Φ_s_ and Φ_tot_ were higher in Q4 vs Q1 (Table 6). In contrast, the DI was reduced in Q4 vs Q1 (Table 6). Compared with Q1, insulin clearance was lower in Q2–4 (Table 6). Individuals in Q4 also exhibited reduced insulin clearance in comparison with Q2 and Q3 (Table 6). Modest changes in the amino acid profile were noted, with aspartate being higher in Q4 vs Q1 and Q2, and glutamate was increased in Q4 vs Q2 (Table 7).

**Table 5:** Sex-specific normoglycaemic participant characteristics

Characteristic	Women				Men			
	Q1	Q2	Q3	Q4	Q1	Q2	Q3	Q4
*n*	33	33	32	32	22	23	23	23
Age (years)	23 (8)	24 (5)	22 (3)	23 (3)	24 (9)	24 (5)	23 (3)	27 (8)^‡^
Height (cm)	168 ± 1	167 ± 1	165 ± 1	162 ± 1*	182 ± 1	180 ± 1	178 ± 1	178 ± 1
Weight (kg)	64.3 ± 1.5	63.5 ± 1.9	61.7 ± 1.5	60.4 ± 1.6	81.7 ± 1.8	81.0 ± 2.4	77.8 ± 2.7	79.8 ± 2.5
BMI (kg/m^2^)	22.8 ± 0.5	22.8 ± 0.5	22.6 ± 0.5	23.0 ± 0.6	24.7 ± 0.5	25 ± 0.6	24.4 ± 0.6	25.2 ± 0.8
BMI > 25 kg/m^2^ (%)	21	21	19	33	36	39	39	44
Body fat (%)^a^	27.8 ± 1.0	29.4 ± 1.0	31.8 ± 1.5	34.2 ± 1.5*	15.0 ± 0.9	16.9 ± 1.1	19.9 ± 1.5*	26.5 ± 1.6*^†‡^
Fat mass (kg)	17.2 (5.9)	16.9 (6.3)	17.9 (8.8)	21.6 (8.0)	11.3 (4.8)	13.1 (7.7)	15.5 (8.9)	21.3 (10.6)*
Visceral fat (g)	46.5 (75)	82.0 (119.3)	49.5 (194.0)	60.5 (326.3)	299 (78)	207 (246)	188 (171)	301 (800)
Fat-free mass (kg)	44.2 ± 1.1	42.2 ± 1.2	39.7 ± 1.1*	38.3 ± 1.3*	65.8 ± 1.7	64.5 ± 2.7	56.9 ± 2.1*^†^	55.4 ± 1.8*^†^
SBP	115 ± 2	117 ± 3	115 ± 2	117 ± 3	126 (15)	126 (12)	120 (15)	124 (13)
DBP	70 (10)	76 (11)	75 (13)	80 (14)	74 ± 2	75 ± 2	75 ± 2	81 ± 2
Fasting glucose (mmol/l)	5.3 (0.6)	5.4 (0.5)	5.2 (0.6)	5.3 (0.6)	5.3 ± 0.1	5.5 ± 0.1	5.3 ± 0.1	5.5 ± 0.1
2 h glucose (mmol/l)	4.9 (1.6)	5.3 (1.2)	5.3 (1.4)	5.9 (2.0)*	4.3 ± 0.3	4.8 ± 0.3	5.1 ± 0.3	5.3 ± 0.2
Fasting insulin (pmol/l)	13.0 (9.8)	16.7 (15.4)	20.5 (11.7)*	31.4 (15.8)*^†^	8.0 (9.1)	11.8 (7.8)	20.1 (11.8)*	31.5 (38.5)*^†^
2 h insulin (pmol/l)	93.2 (42.3)	142.3 (79.0)*	186.4 (65.2)*^†^	262.2 (105.3)*^†‡^	27.7 (24.0)	72.3 (60.7)	118.8 (77.1)*	240.1 (319.3)*^†^
Fasting C-peptide (pmol/l)	263.7 (151.7)	246.0 (145.5)	290.7 (142.5)	314.6 (190.9)	165.1 (49.7)	242.3 (82.6)	305.9 (183.5)*	406.4 (257.7)*^†^
Fasting NEFA (mmol/l)	0.24 (0.13)	0.26 (0.11)	0.22 (0.13)	0.26 (0.22)	0.21 (0.17)	0.18 (0.10)	0.24 (0.26)	0.22 (0.10)
Fasting triacylglycerol (mmol/l)	0.66 (0.19)	0.70 (0.27)	0.73 (0.37)	0.83 (0.30)	0.61 (0.17)	0.59 (0.28)	0.76 (0.23)	0.87 (0.27)*^†^
Total cholesterol (mmol/l)	3.9 (1.0)	4.1 (1.1)	4.5 (0.7)	4.3 (1.1)	3.8 (0.7)	3.9 (0.8)	3.7 (0.8)	4.2 (1.0)
HDL-cholesterol (mmol/l)	1.5 (0.4)	1.4 (0.5)	1.5 (0.4)	1.6 (0.5)	1.5 (0.2)	1.3 (0.3)	1.3 (0.4)	1.3 (0.5)
Non-HDL-cholesterol (mmol/l)	2.4 (0.6)	2.5 (0.9)	2.9 (0.9)	2.7 (1.1)	2.2 (0.5)	2.4 (0.7)	2.3 (0.7)	2.8 (1.2)
Family history of diabetes (*n*)	8	10	8	11	4	2	8	5
Smokers (*n*)	2	1	0	2	1	2	0	0
Energy intake (MJ)^b^	9.1 ± 0.4	7.7 ± 0.4	8.1 ± 0.4	7.4 ± 0.4*	11.8 (5.7)	12.1 (3.9)	9.3 (2.8)	10.6 (4.7)
% carbohydrate	37.3 (8.7)	41.1 (8.4)	40.3 (7.9)	41.6 (11.3)	36.0 ± 3.4	39.8 ± 2.4	40.1 ± 3.0	42.4 ± 2.7
% protein	19.6 (6.3)	18.0 (3.5)	19.0 (4.7)	18.4 (3.3)	22.2 (8.9)	19.3 (5.4)	19.1 (8.1)	20.1 (6.2)
% fat	35.6 ± 1.1	34.1 ± 1.4	36.0 ± 1.3	34.5 ± 1.5	36.5 (19.0)	34.3 (6.7)	33.0 (12.5)	36.1 (6.8)
Contraceptive use (*n*)	3	5	7	10	–	–	–	–
White (*n*)	29	26	25	23	21	23	17	11
Asian (*n*)	3	7	7	8	1	0	6	11
Other (*n*)	1	0	0	1	0	0	0	1

Data are mean ± SEM, median (interquartile range) or proportion (%) as appropriate

*p < 0.05 vs Q1

^†^*p* < 0.05 vs Q2

^‡^*p* < 0.05 vs Q3

^a^Data on body composition (percentage fat, fat mass, visceral fat and lean mass) are from *n* = 26 for Q1, *n* = 28 for Q2, *n* = 24 for Q3 and *n* = 18 for Q4 for women and *n* = 20 for Q1, *n* = 14 for Q2, *n* = 16 for Q3 and *n* = 12 for Q4 for men

^b^Data on energy and macronutrient intake are from *n* = 25 for Q1, *n* = 28 for Q2, *n* = 24 for Q3, *n* = 23 for Q4 for women and *n* = 16 for Q1, *n* = 18 for Q2, *n* = 17 for Q3 and *n* = 10 for Q4 for men

**Table 6:** Sex-specific S_I_, GE and beta cell function in normoglycaemic participants

Variable	Women				Men			
	Q1	Q2	Q3	Q4	Q1	Q2	Q3	Q4
S_I_^D^ (dl^−4^ kg^−1^ min^−1^ [pmol/l]^−1^)	226.0 (149.1)	142.9 (64.9)*	104.7 (101.9)*	67.3 (66.9)*^†‡^	382.6 (215.1)	217.8 (136.3)	118.8 (67.0)*^†^	53.3 (66.2)*^†^
S_I_^L^ (dl^−4^ kg^−1^ min^−1^ [pmol/l]^−1^)	176.6 (116.4)	87.2 (51.6)*	66.3 (38.2)*	44.3 (28.7)*^†^	225.1 (135.1)	166.1 (97.0)	86.2 (36.9)*^†^	50.7 (59.6)*^†^
S_I_ (dl^−4^ kg^−1^ min^−1^ [pmol/l]^−1^)	430.0 (274.1)	225.0 (102.6)*	168.2 (114.3)*	115.7 (85.5)*^†^	582.6 (361.0)	350.1 (129.3)	203.7 (101.5)*^†^	116.6 (105.0)*^†^
GE^D^ (dl kg^−1^ min^−1^)	0.027 (0.006)	0.025 (0.004)	0.025 (0.005)	0.025 (0.003)*	0.027 (0.007)	0.027 (0.005)	0.026 (0.005)	0.025 (0.002)
GE^L^ (dl kg^−1^ min^−1^)	0.016 (0.026)	0.027 (0.029)	0.023 (0.037)	0.025 (0.035)	0.018 (0.018)	0.022 (0.029)	0.014 (0.014)	0.023 (0.020)
GE (dl kg^−1^ min^−1^)	0.043 (0.024)	0.052 (0.031)	0.050 (0.032)	0.050 (0.037)	0.045 (0.023)	0.049 (0.029)	0.042 (0.015)	0.049 (0.019)
Φ_b_ (10^−9^ min^−1^)	3.9 (2.6)	3.3 (2.3)	4.3 (2.3)	4.3 (2.3)	2.2 (0.7)	3.2 (1.2)	3.6 (3.4)*	4.9 (3.7)*^†^
Φ_d_ (10^−9^)	342 (223)	330 (417)	436 (367)	484 (422)	195 (147)	277 (334)	316 (406)*	449 (524)*
Φ_s_ (10^−9^ min^−1^)	30.5 (13.4)	37.8 (17.2)	39.1 (26.9)	52.0 (23.3)*	34.2 (17.6)	36.7 (30.5)	48.4 (24.6)	49.8 (35.8)
Φ_tot_ (10^−9^ min^−1^)	32.1 (13.1)	38.5 (18.0)	40.3 (26.5)	53.7 (25.0)*	35.3 (17.8)	36.8 (31.7)	48.7 (24.4)	52.5 (39.1)*
DI (10^−14^ dl kg^−1^ min^−2^ [pmol/l]^−1^)	2644 (2514)	2048 (1259)	1655 (2126)	1129 (1618)*	4439 (2944)	2713 (2773)	2022 (1338)*	1128 (1384)*^†^
Insulin clearance (l min^−1^ m^−2^)	1.7 (0.9)	1.3 (0.4)*	1.0 (0.5)*	0.8 (0.4)*^†‡^	1.7 (0.5)	1.2 (0.3)	1.1 (0.4)*	0.7 (0.4)*^†‡^

Data are median (interquartile range)

*p < 0.05 vs Q1

^†^*p* < 0.05 vs Q2

^‡^*p* < 0.05 vs Q3

**Table 7:** Sex-specific normoglycaemic plasma amino acid profiles

Amino acid (μmol/l)	Women				Men			
	Q1	Q2	Q3	Q4	Q1	Q2	Q3	Q4
Alanine	291.2 (66.6)	285.2 (50.2)	301.8 (88.9)	294.2 (97.7)	256.6 (53.2)	281.2 (41.5)	284.1 (99.2)	325.5 (101.2)*
Glycine	221.8 ± 11.4	229.3 ± 10.5	224.2 ± 10.0	221.7 ± 11.5	223.8 (52.9)	227.7 (46.0)	227.6 (32.8)	224.0 (58.7)
Valine	203.3 (43.6)	194.8 (35.7)	200.9 (52.2)	202.7 (51.0)	244.0 ± 9.8	244.6 ± 8.4	245.5 ± 10.3	265.7 ± 10.9
Leucine	106.6 (20.5)	104.0 (19.5)	103.7 (29.3)	106.1 (30.5)	132.5 (19.1)	129.6 (20.3)	129.0 (27.7)	141.4 (25.5)
Isoleucine	50.8 (10.0)	49.9 (11.7)	51.3 (12.5)	52.5 (16.9)	62.5 (10.2)	62.4 (9.7)	61.1 (12.3)	71.3 (11.2)^†^
Proline	127.9 (42.9)	127.5 (70.1)	143.9 (39.1)	147.2 (52.1)	152.9 (49.4)	186.1 (61.2)	182.9 (64.0)	190.4 (93.1)*
Methionine	21.9 (4.2)	20.9 (5.6)	22.0 (5.7)	23.2 (4.3)	25.3 (4.6)	22.7 (7.5)	26.3 (6.3)	25.6 (8.2)
Serine	100.7 ± 2.8	103.4 ± 3.2	105.7 ± 3.5	104.6 ± 3.3	98.5 ± 4.2	95.9 ± 1.8	100.7 ± 3.5	104.1 ± 3.6
Threonine	120.3 (53.3)	128.4 (47.8)	162.0 (84.6)	141.3 (68.9)	115.5 ± 6.4	138.6 ± 5.9	131.7 ± 6.1	147.6 ± 7.8*
Phenylalanine	56.1 (7.4)	55.0 (11.9)	57.3 (13.7)	59.4 (9.7)	59.9 (9.1)	60.5 (7.9)	61.4 (12.1)	66.8 (11.3)
Aspartate	1.8 (1.4)	2.0 (1.6)	2.8 (2.4)	3.3 (5.1)*^†^	1.4 (1.3)	4.9 (6.6)	2.6 (3.7)	4.3 (3.5)*
Glutamate	22.0 (30.2)	18.4 (14.6)	19.5 (22.2)	34.2 (68.5)^†^	23.3 (16.1)	31.1 (66.1)	35.1 (39.5)	55.3 (84.4)*
Glutamine	449.4 (86.5)	492.1 (90.1)	441.2 (98.7)	454.7 (58.3)	513.6 (55.3)	510.1 (152.6)	527.3 (80.0)	503.9 (119.2)
Tyrosine	56.7 (18.7)	56.0 (19.4)	61.0 (22.9)	58.3 (15.0)	59.6 (13.4)	69.9 (26.3)	67.8 (23.2)	80.2 (32.4)*
Total BCAA	358.2 (68.6)	354.0 (68.5)	353.2 (98.5)	359.9 (83.1)	429.1 (85.0)	422.0 (78.8)	431.6 (89.6)	479.2 (98.1)

Data are mean ± SEM or median (interquartile range)

*p< 0.05 vs Q1

^†^*p*< 0.05 vs Q2

For men, those in Q4 were older than those in Q3 but all quartiles had similar height, weight and BMI (Table 5). Q4 exhibited the highest percentage body fat (Table 5), while fat-free mass was lowest in Q3 and Q4 compared with both Q1 and Q2 (Table 5). Plasma triacylglycerols were elevated in Q4 compared with Q1 and Q2 (Table 5). While family history of diabetes was similar across quartiles, there was a greater proportion of Asians in Q4 than in the other quartiles (Table 5). Fasting (Table 5) and OGTT (Fig. 3b, c) glucose responses were similar across quartiles, yet there was a progressive increase in insulin under both fasting (Table 5) and OGTT conditions (Fig. 3e, f). In contrast, C-peptide responses were higher in Q2–4 vs Q1 (Fig. 3h, i). ISRs were higher in Q3 and Q4 vs Q1 and Q2 (Fig. 3k, l). All indices of S_I_ were lower in Q3 and Q4 vs Q1 and Q2 (Table 6). Compared with Q1, both Φ_b_ and Φ_d_ were higher in individuals in Q3 and Q4 (Table 6). Individuals in Q4 also had higher Φ_b_ compared with those in Q2 (Table 5). Φ_tot_ was elevated in individuals in Q4 vs those in Q1 (Table 6). The DI progressively decreased across quartiles such that the DI of Q3 was lower than Q1, and Q4 was lower than Q1 and Q2 (Table 6). A reduction in insulin clearance was evident between Q4 and all other quartiles (Table 6). In addition, insulin clearance in Q3 was lower than that in Q1 (Table 6). A number of amino acids including alanine, proline, threonine, aspartate, glutamate and tyrosine were elevated in Q4 compared with Q1, while isoleucine was higher in Q4 than in Q2 (Table 7).

### Relationship among body composition, insulin responses and S_I_

For both sexes, insulin AAB was positively correlated with percentage body fat and fat mass, but negatively correlated with fat-free mass (Table 8). Similar associations were found in the whole cohort, with the addition of a negative correlation with body weight (Table 8). Visceral fat only correlated with the insulin response in women. Total S_I_ negatively correlated with BMI, percentage fat and fat mass in women, while in men the only significant negative correlation was with percentage body fat (Table 8). In the combined cohort, a negative correlation was found between S_I_ and percentage fat and fat mass, while fat-free mass was positively associated with S_I_ (Table 8). Note, the strengths of all correlations were for the most part relatively modest.

**Table 8:** Correlations among body composition, OGTT insulin responses and S_I_

Variable	Insulin AAB			S_I_		
	Women	Men	Combined	Women	Men	Combined
Body weight	−0.11	0.01	−0.26***	−0.06	−0.05	0.09
BMI	0.05	0.09	−0.05	−0.17*	−0.17	−0.18
% body fat	0.40***	0.62***	0.57***	−0.34***	−0.28*	−0.49***
Fat mass	0.23*	0.49***	0.41***	−0.30**	−0.20	−0.44***
Visceral fat	0.26**	0.05	0.01	−0.04	0.03	−0.13
Fat-free mass	−0.38***	−0.43***	−0.48***	0.09	0.23	0.32***

Data are Spearman *r* correlation coefficients

*p < 0.05

**p < 0.01

***p < 0.001

Insulin AAB, AAB for the integrated insulin response during the OGTT

## Discussion

We aimed to develop a deeper understanding of the systems-level mechanisms involved in obesity-independent hyperinsulinaemia. By comparing the phenotype of the normoglycaemic insulin response quartiles, it was apparent that hyperinsulinaemia manifested not only as a result of heightened insulin secretion but also due to reduced insulin clearance. Postprandial hyperinsulinaemia was also associated with a reduction in both disposal and liver S_I_. Thus, the combined actions of reduced S_I_, enhanced insulin secretion and reduced insulin clearance appear to contribute to postprandial hyperinsulinaemia in young non-obese adults. Our data are consistent with recent findings whereby reduced insulin clearance was identified as an early adaption to impaired S_I_ [15], a response likely important for preserving beta cell function by minimising the insulin secretory burden associated with insulin resistance [30]. Furthermore, despite similar glycaemic responses, and in the face of increased insulin secretion, beta cell function declined across quartiles evidenced by the ~65% reduction in DI between Q1 and Q4. This is similar to findings of Ferrannini et al. [31], where a reduction in beta cell function within the normal glucose tolerance range was associated with rising 2 h glucose concentrations. This may explain why Q4 exhibited the highest 2 h glucose levels of all quartiles. Importantly, these findings were largely similar when comparing the whole cohort or examining women and men separately.

Despite studying a non-obese relatively narrow BMI range, we observed that increased adiposity (percentage fat and total fat mass) was associated with heightened insulin responses and reduced S_I_ in the entire cohort, and in both sexes. Interestingly, visceral fat only correlated positively with the OGTT insulin response in women. This suggests that within this relatively narrow BMI range, percentage body fat and absolute fat mass, but not visceral fat, are the strongest predictors of the insulin response and S_I_. This provides additional evidence, to support previous findings [32, 33], that visceral fat is not necessarily associated with hyperinsulinaemia and insulin resistance.

The progressive hyperinsulinaemia across quartiles could be due to primary insulin hypersecretion, compensation for insulin resistance or a combination of both. Unfortunately, due to the cross-sectional nature of our work, this distinction could not be determined. However, there is strong evidence in animals [34–40] and humans [11–13, 41] to support a causal role of excess insulin in driving insulin resistance, and that suppression of high plasma insulin levels enhances insulin action [42–44]. In this regard, it is of interest to note the recent work of Tricò et al. [8], which suggests that hyperinsulinaemia can arise due to primary insulin hypersecretion independent of insulin resistance. With this in mind, further studies examining the role of primary insulin hypersecretion vs primary insulin resistance in humans, while difficult, are warranted to address this fundamental problem [9]. Ascertaining the major pathways and organs initiating hyperinsulinaemia would pave the way for deciphering molecular mechanisms driving this phenomenon, including epigenetic modifications given the heritable nature of metabolic dysfunction [45].

While it was not our intention to screen for prediabetes prevalence, we identified a significant proportion of individuals exhibiting IFG (5.5%) and IGT (7.5%). This allowed us to investigate what may contribute to the transition from the normoglycaemic hyperinsulinaemic state to the prediabetic condition by comparing the Q4 phenotype with that of IFG and IGT. This revealed that individuals in Q4 were more similar to IGT than IFG. In fact, despite similar glycaemic responses during the OGTT, insulin levels were markedly higher in Q4 than in IFG. S_I_, particularly in relation to glucose production, and insulin clearance were also lower in Q4 than in IFG. Although the magnitude of the postprandial insulin response in Q4 and in IGT was similar, the dynamics were different. Individuals with IGT exhibited a delayed insulin peak subsequently resulting in higher 2 h insulin levels than in Q4. Furthermore, the most dramatic reductions in S_I_ were observed in individuals within Q4 and with IGT. Although not statistically different, both disposal and production S_I_ were somewhat lower in individuals with IGT than in individuals in Q4, likely due to the exacerbation of insulin resistance by postprandial hyperglycaemia [46]. The key distinguishing feature, however, was the severely impaired beta cell function in individuals with IGT, evidenced by the dramatic reduction in DI when compared with individuals in Q4. This provides further evidence that postprandial hyperglycaemia develops when beta cell insulin secretion and reduced insulin clearance can no longer compensate for insulin resistance. With respect to individuals in Q4, we can only speculate in terms of future metabolic outcomes, but it seems that even though these individuals had sufficient beta cell function to maintain normoglycaemia, any subsequent decline in insulin secretion capacity could quickly precipitate IGT. In addition, plasma triacylglycerols and cholesterol were highest in Q4, albeit within the normal range, which may also increase future cardiovascular disease risk.

An amino acid signature of insulin resistance suggests that elevated BCAAs are associated with impaired S_I_ [24–26]. Furthermore, a Mendelian randomisation study suggests that insulin resistance causally increases plasma BCAAs [25]. We did not detect any progressive increase in either the individual or total BCAAs across insulin response quartiles in the entire cohort. The discrepancies among studies may be explained by the demographics of the participants, with our cohort being younger and having a lower BMI. Nonetheless, this has implications for the use of plasma BCAAs as biomarkers since this may not be effective in identifying insulin resistance in young non-obese adults. In contrast, a number of amino acids including phenylalanine, aspartate, glutamate and glutamine were modestly elevated in the most hyperinsulinaemic quartile. Sex-specific comparisons also revealed that men in Q4 exhibited small increases in alanine, isoleucine, proline, threonine, aspartate, glutamate and tyrosine. The physiological significance and underlying mechanisms responsible are unclear. Nonetheless, these findings support the suggestion of sex-specific regulation of amino acid metabolism [47].

There are some limitations with this study. First, body composition data were not available for the entire cohort. Despite ~28% of individuals having missing data, we were able to demonstrate the well-known link among increased adiposity, hyperinsulinaemia and reduced S_I_. Also, physical activity or fitness was not assessed. It is possible that the most hyperinsulinaemic individuals were less physically active, contributing to their high degree of insulin resistance. Although individuals were asked to refrain from exercise 48 h prior to testing, it cannot be ruled out that any residual insulin-sensitising effect of exercise conducted before this period may have influenced our results. Finally, we did not document or control for menstrual cycle phase. The impact of this on our findings is uncertain as there is conflicting evidence regarding the effect of menstrual cycle phase on S_I_ [48–50].

In conclusion, we show that in non-obese young adults hyperinsulinaemia is not simply caused by insulin hypersecretion; rather, it arises due to the combined effects of increased insulin secretion and markedly reduced insulin clearance. A reduction in insulin clearance should therefore be viewed as an early driver of hyperinsulinaemia in young non-obese adults. Of concern, a significant proportion of our cohort had prediabetes (either IGT or IFG). Interestingly, the metabolic phenotype of the most hyperinsulinaemic normoglycaemic quartile was more similar to IGT than IFG, with this quartile also exhibiting the highest plasma triacylglycerols. This may indicate that hyperinsulinaemic individuals are more likely to transition to IGT than IFG, and may be at greater risk of developing manifestations of the metabolic syndrome. Together, these findings provide insight into the metabolic abnormalities that occur across the continuum of insulin responses through the spectrum of normoglycaemia and prediabetes independent of obesity. Ultimately, this may help define the factors that underlie the transition from the normoglycaemic hyperinsulinaemic insulin-resistant state to the prediabetic condition.

## Data Availability

The datasets generated during and/or analysed during the current study are available from the corresponding author on reasonable request.

## Abbreviations

AAB: Area above basal
BCAA: Branched-chain amino acid
DI: Disposition index
GE: Glucose effectiveness
GE^D^: Ability of glucose to stimulate glucose disposal
GE^L^: Ability of glucose to inhibit glucose production
IFG: Impaired fasting glucose
IGT: Impaired glucose tolerance
ISR: Insulin secretion rate
Q: Quartile
S_I_: Insulin sensitivity
S_I_^D^: Ability of insulin to stimulate glucose disposal
S_I_^L^: Ability of insulin to inhibit glucose production
Φ_b_: Basal beta cell responsivity
Φ_d_: The dynamic component of beta cell responsivity
Φ_s_: The static component of beta cell responsivity
Φ_tot_: Total beta cell responsivity
